# Inconsistencies in the drawing and interpretation of smiley faces: an observational study

**DOI:** 10.1186/s13104-018-3185-0

**Published:** 2018-01-27

**Authors:** Mike Clarke, Helen McAneney, Fiona Chan, Lisa Maguire

**Affiliations:** 1 Northern Ireland Methodology Hub, Centre for Public Health, Queen’s University Belfast, Belfast, Northern Ireland BT12 6BJ UK; 2 MRC North West Hub for Trials Methodology Research, University of Liverpool, Liverpool, UK

**Keywords:** Smiley faces, Drawing, Draw-A-Man Test, Facial recognition

## Abstract

**Objectives:**

Pre-prepared smiley face symbols are used widely to gather information on, for example, satisfaction with services or health and well-being. We investigated how women and men of different ages respond when asked to draw a smiley face for themselves. Our objectives were to investigate how they differ by generating a unique set of data to explore this simple human behaviour and to illustrate the importance of considering gender and age mix in any study.

**Results:**

We collected 723 drawings, in a variety of settings. Gender and age were provided for 676 drawings (women: 511; men: 165; ≤ 30 years: 335; > 30 years: 341). Although similar proportions of women and men drew some features, such as closed mouths; women and those aged ≤ 30 were less likely to draw noses and outlines around the faces, and more likely to draw a classic smiley face. Our analyses provide a novel way to highlight that whenever self-reported outcomes are compared between groups, the group composition for characteristics such as gender and age may need to be considered carefully to explore whether differences in outcomes might simply arise from imbalances in those characteristics.

## Introduction

Whether it’s a baby looking at its parents [1], a glance of someone we think we know, a study of the prevalence of moustaches in medical leaders [2] or a computer allowing us access into a country [3], facial recognition is common in our daily lives. Furthermore, patients and the public are increasingly prompted to provide feedback on their health and well-being or their satisfaction with services by selecting from a series of faces, at least one of which will be smiling.

These images are usually pre-prepared but we wished to investigate whether there would be differences in how women, men and different age groups respond when asked to draw a smiley face for themselves. This allowed us to investigate the question of how different genders and age groups would complete this simple task and to provide a novel way to show that if differences do exist between genders or age groups, this might suggest a need to consider such factors carefully in instances such as questionnaire design and analysis. This might be necessary, for instance, when surveys use scales that incorporate a range of faces (from happy through neutral to sad looking), or when something such as the Wong-Bakes scale is used for pain rating using facial images [4]. If these are not gender or age neutral for gathering data, variation in these facial pictures (e.g. the inclusion of a boundary; circles, dots or dashes for eyes, etc.) might bias the responses received. Exploring what people regard as the key features of a face, allows one to consider the implications of any potential for bias and, consequently, the interpretation of data gathered using facial prompts. Therefore, we investigated these issues in a large observational study in which participants at a variety of events were asked to draw a smiley face. Although this does not necessarily reveal how different groups would react to different pre-prepared images, it provides insight into what they themselves would regard as a smiley face.

The use of drawing within health research is not new. For example, the Drawing a Man test by Goodenough in 1926 provided a score based on the features drawn by children aged 4–10 years as a means to assess the child’s intelligence. The human figures drawn were scored based on the presence and number of details in the drawing, as well as the accuracy of placement of each body part [5]. The test has been revised and extended, with the 2004 version by Reynolds and Hickman requiring children, adolescents and adults to draw themself [6].

In this paper, we report results from the Smiley Faces study, an observational study of how different types of people draw smiley faces, exploring the differences between genders and ages. We believe that this is the first study of its kind, although there are earlier examples of research into the relationship between, for example, age and mental health and the drawing of human figures [7]. Our objectives were to investigate how women, men and different age groups differ in the simple task of drawing a smiley face by generating a unique set of data to explore this simple human behaviour and to illustrate the importance of considering gender and age mix in any study.

## Main text

### Methods

**Fig. 1:**
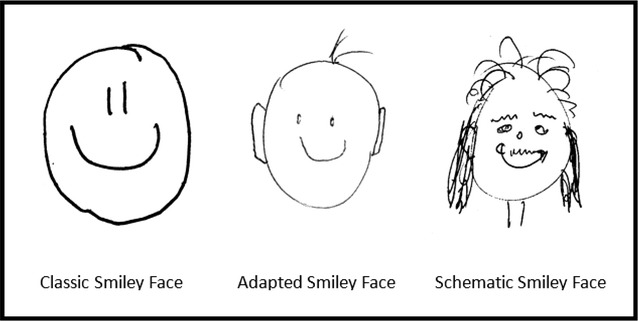
Examples from the drawings produced as part of the smiley faces study

We collected data for the Smiley Faces study at a variety of conferences, meetings and teaching sessions, partly as a tool to illustrate key points in the design of clinical trials [8]. There was heterogeneity across these settings in relation to other aspects of the events, but we standardised our activity and presented it in a serious manner each time. All participants were first asked to write the title and date of the relevant event on a piece of paper. They were then told that they would be given a simple instruction, which would be said once and would not be expanded upon or explained, before being given the instruction: “draw a smiley face”. Participants usually took less than 1 min to do their drawing and were then asked to write their name, age and gender on the paper, if they wished to do so, before the drawings were collected. Although it was suggested at the end of some of the sessions that the use of the term “smiley face” might lead everyone to produce a classic smiley face, as originally drawn by Harvey Ball in 1963 [9], we always used this same instruction to standardise it across the events. And, as shown below, a variety of faces were drawn (see Fig. 1).

Drawings were coded to categorise the faces and to record the presence of specific features. The faces were categorised as one of the following:

- Classic smiley face: dots or dashes representing eyes and a line for a closed mouth (with or without an outline around the facial features).
- Adapted smiley face: a classic smiley face with added features, such as hair.
- Schematic face: a more natural representation of a face.

Specific features on each drawing were coded as present or absent, consistent with the scoring system developed by Goodenough for drawings of the whole body [5]. In the Goodenough system, an outline of the head, the presence of eyes, nose, mouth, hair, and ears, as well as further details of the eyes such as brow, lashes, and the pupil, each receive a point if present. In our system, we also recorded additional items and fine details. Specifically, we recorded the presence of elements such as a defined outline for the face, eyes, mouth, nose, cheeks, chin, ears, hair, eyebrows [10], freckles, teeth, tongue, eyelashes, moustaches [2], and other features (e.g. glasses or hat). Finer detail was recorded for some of these elements to distinguish, for example, open and closed mouths; and the use of dots, open or filled circles, dashes or richer depictions (e.g. with irises and pupils) for eyes. The coding was done by two authors (FC and LM) independently, with discrepancies resolved by consensus between them. No disagreements remained after these discussions. If there had been discrepancies, the other authors would have determined the final coding.

The coded data were analysed using SPSS (v22) giving a combination of descriptive and comparative statistics: principally, odds ratio (OR) and the associated 95% confidence intervals (CI) to investigate differences between the participant subgroups. The pre-specified analyses compared drawings by women versus men and between different age groups (determined by dividing the data into two similarly sized age groups) for:

- Category of face
- Presence of a nose
- Type of mouth
- Outline around the face.

### Results

From 2013 to 2016, a total of 723 drawings were collected at events in Canada, England, Ireland, Italy, and Northern Ireland; overseen by two authors (MC and HM). The events ranged from less than 10 participants in each of several small group teaching sessions, to 136 in a lecture to nursing and midwifery students in Ireland. Using the baseline characteristics of the participants as given by the information they wrote on the drawings, the total sample included more women than men (530 vs. 175) and dividing the data at age 30 years produced similar numbers of people below (335) and above (341) this age. Data for the 676 participants who provided both age and gender were used for the analyses presented in this paper (women: 511; men: 165; ≤ 30 years of age: 335; > 30 years of age: 341). This excludes 29 drawings with gender but not age, and 18 with neither age nor gender.

Among the 676 included drawings, there were 371 (54.9%) classic smiley faces, 273 (40.4%) adapted smiley faces and 32 (4.7%) schematic faces (see Fig. 1 for illustrative examples). Considering the specific features for all participants combined, a minority drew a nose (261, 38.6%), most drew a closed mouth (572, 84.6%) and most drew an outline around the features (479, 70.9%) (Table 1). Some of the rarest features were a chin (1, 0.1%), freckles (2, 0.3%), a moustache (3, 0.4%) and a neck (6, 0.9%).

**Table 1:** Coding of the drawings

Coding^a^	Frequency (%)
Classic face	371 (54.9)
Adapted face	273 (40.4)
Schematic face	32 (4.7)
Nose present	261 (38.6)
Nose absent	415 (61.4)
Closed mouth	572 (84.6)
Open/other mouth	102 (15.1)
No mouth	2 (0.3)
Outline present	479 (70.9)
Outline absent	197 (29.1)

^a^A total of 676 participants were included in the analysis, having provided both age and gender. The remaining 47 (6.5%) drawings were excluded because age and or gender were missing

There were some significant differences in our primary comparisons (Table 2). Women were much less likely than men to draw noses (OR: 0.55, 95% CI: 0.38–0.78, p = 0.0008) and outlines around the face (OR: 0.55, 95% CI 0.36–0.84, p = 0.006), but much more likely to draw a classic smiley face (OR: 1.95, 95% CI 1.36–2.78, p = 0.0002). On the other hand, similar proportions of women and men drew some features, such as closed mouths (84.9% vs. 83.6%).

**Table 2:** Coding of facial features by gender and age of the drawer

	Total	Gender		Odds ratio (95% CI), *p* value
		Male	Female	
All drawings	676	165	511	–
Nose present	261 (38.6%)	82 (49.7%)	179 (35.0%)	0.55 (0.38–0.78), 0.0008
Closed mouth	572 (84.6%)	138 (83.6%)	434 (84.9%)	0.91 (0.56–1.46), 0.688
Outline present	479 (70.9%)	131 (79.4%)	348 (68.1%)	0.55 (0.36–0.84), 0.006
Classic face	371 (54.9%)	70 (42.4%)	301 (58.9%)	1.98 (1.36–2.78), 0.0002

	Total	Age		
		≤ 30 years	> 30 years	
All drawings	676	335	341	–
Nose present	261 (38.6%)	97 (29.0%)	164 (48.1%)	0.44 (0.32–0.60), < 0.0001
Closed mouth	572 (84.6%)	286 (85.4%)	286 (83.9%)	1.12 (0.74–1.71), 0.588
Outline present	479 (70.9%)	199 (59.4%)	280 (82.1%)	0.32 (0.22–0.45), < 0.0001
Classic face	371 (54.9%)	225 (67.2%)	146 (42.8%)	2.73 (2.00–3.74), < 0.0001

People aged 30 years and under (both men and women) were less likely than older participants to draw noses (OR for both genders combined: 0.44, 95% CI 0.32–0.60, p < 0.0001) and outlines around the face (OR for both genders combined: 0.32, 95% CI 0.22–0.45 p < 0.0001), and more likely to draw a classic smiley face (OR for both genders combined: 2.73, 95% CI 2.00–3.74, p < 0.0001) (Table 2). These relationships with age are similar for men and women.

### Discussion

Our study revealed interesting differences and similarities between the types of participant in how they interpret the instruction to draw a smiley face and the features that they include in their drawings. This variety may be due to the characteristics of the participants, bearing in mind that we found significant differences between women and men, and between younger and older participants. Women, and those aged 30 years or younger were more likely to draw a classic smiley face, and to avoid drawing both noses and outlines around the faces.

On the dozens of occasions that we have conducted the Smiley Faces study, it has generated a variety of interpretations and conclusions amongst those who did the drawings, and we are grateful to them for their insights and suggestions. With this analysis, we are able to confirm some of the differences, but not necessarily explain them and they generate many questions that may be relevant when faces are used to gather health or other information. For example, do women pay less attention to fine detail, such as noses, than men? Are men more constrained in their thinking than women and less of a “free spirit” because they draw their faces with an outline around, setting a boundary? Should these be seen as positive or negative traits in the types of people who have taken part in the Smiley Faces study?

In today’s society, facial recognition is used in many areas of our lives. Yet little research is available on how different people draw or interpret faces. Our analyses support some of the interpretations suggested by participants in the dozens of events at which we have conducted this study, but do not necessarily explain them. However, they do show that if such drawings are to be used in research or in psychological assessments (such as with the Goodenough’s Draw a Man Test), or for obtaining feedback on satisfaction, health or well-being, possible differences by gender and age may need to be considered when gathering, interpreting and comparing data from different settings. The Smiley Faces study has also used a novel approach to highlight the general point that whenever self-reported outcomes are compared between groups, the composition of those groups in relation to characteristics such as gender and age might need to be considered carefully to ensure that any differences in outcomes are not simply due to imbalances in those characteristics.

## Limitations

We realise that the differences we found between genders and age groups may be a result of confounding, which is not within our ability to investigate, but the findings are of interest. The participants were all given the same simple task but it produced a wide variety of different drawings. The reasons for the differences may never truly be known or understood, but this study reveals how people may unknowingly interpret day-to-day tasks differently.
